# Patient-reported outcomes after chemoradiotherapy for anal cancer

**DOI:** 10.2340/1651-226X.2025.43636

**Published:** 2025-07-30

**Authors:** Kathinka Schmidt Slørdahl, Eva Skovlund, Jan-Åge Olsen, Ragnhild Tvedt, Maria Thomsen, Stein Kaasa, Marianne Grønlie Guren

**Affiliations:** a Department of Oncology, Oslo University Hospital, Oslo, Norway; b Institute of Clinical Medicine, Faculty of Medicine, University of Oslo, Oslo, Norway; c Department of Public Health and Nursing, Norwegian University of Science and Technology (NTNU), Trondheim, Norway; d European Palliative Care Research Centre (PRC), Department of Oncology, Oslo, Norway

**Keywords:** Anal cancer, chemoradiotherapy, patient-reported outcome measures, chronic fatigue, anorectal function

## Abstract

**Background and purpose:**

Chemoradiotherapy (CRT) for squamous cell carcinoma of the anus (SCCA) results in favorable survival. However, treatment intensity must be balanced against late side effects. The aim of this current study was to prospectively investigate patient-reported outcomes (PROs) before CRT and up to 5 years after completed CRT for SCCA.

**Patient/material and methods:**

This prospective study included 120 patients with SCCA receiving CRT to total doses of 54–58 Gy with concomitant mitomycin and 5-fluorouracil. Patients completed PRO questionnaires before CRT, and at 3 months, 1-, 3-, and 5 years after completed CRT. The questionnaires were the EORTC QLQ-C30 and QLQ-CR29, St. Marks incontinence score, Fatigue Questionnaire, the Hospital Anxiety and Depression Scale, and a scoring for neuroticism.

**Results:**

Patients reported a high burden of symptoms and impaired functional outcomes prior to treatment. Tumor-related symptoms, such as buttock pain, improved (difference 11.1, *p* = 0.002) at a clinically relevant level 3 months after CRT, consistent with tumor response. Other functional outcomes and symptoms, such as body image (difference 11.5, *p* < 0.001), worsened. While some outcomes, such as anxiety (difference 10.4, *p* = 0.001), improved over time, several were persistently impaired, in particular anorectal and sexual function, where symptom burden remained high 5 years after CRT. Chronic fatigue (CF) was reported by 28% of patients at 5-year follow-up.

**Interpretation:**

Five years after CRT for SCCA, patients report a persistently high symptom burden regarding anorectal and sexual function, and one-third report CF, demonstrating the long-term impact of treatment.

## Introduction

Chemoradiotherapy (CRT) is the main curative treatment for squamous cell carcinoma of the anus (SCCA) [1]) and has resulted in improved outcomes and lower failure rates in recent years [2]. We have recently reported a 3- and 5-year disease-free survival (DFS) of, respectively, 85 and 78% and a 3- and 5-year overall survival (OS) of, respectively, 93 and 86% in our prospective study [3].

Patient-reported outcome measures (PROMs) provide information about patients’ self-reported health status. Questions often cover areas concerning symptoms, functioning, and health-related Quality of Life (HRQoL). Ideally, the questionnaires should be completed at least at two different times to allow comparisons [4]. PROMs are increasingly used in oncological research, and their importance regarding clinical decisions and in follow-up care is increasingly recognized [5]. Patient-reported outcomes (PROs) are among core outcomes for anal cancer research, and although survival outcomes have been defined, PROs are yet to be defined [6, 7]. Regulatory authorities, including the Food and Drug Administration (FDA) and European Medicines Agency (EMA), now emphasize the inclusion of PRO data in clinical trial reporting as essential for evaluating treatment tolerability [8].

Although CRT results in excellent outcomes in terms of disease control and survival, the disease and treatment have known impact on long-term HRQoL in anal cancer survivors. A previous national study found that anal cancer survivors had significantly impaired HRQoL and higher symptom scores [9] than healthy volunteers. A systematic review by Sodergren et al. found few HRQoL studies in anal cancer patients, most being cross-sectional studies with a low participant number. Diverging results were reported, some studies reported comparable overall HRQoL scores between patients and norms, while others reported impaired overall HRQoL. Impairments on symptom scales regarding urinary-, sexual-, and bowel-related symptoms were common [10]. Late side effects have often been reported as objective physician-graded measures according to Common Terminology Criteria for Adverse Events (CTCAE). A Danish study by Lefèvre et al. compared PROMs and CTCAE and found only fair to moderate agreement between these [11]. This illustrates the importance of prospective PROs studies, following anal cancer patients over time.

Anal cancer is a rare disease [12], but due to high survival rates, there is a considerable number of survivors, potentially having late side effects after treatment. The aim of the current study was to prospectively collect PROMs from start of CRT and up to 5 years after completed CRT for SCCA to investigate the prevalence and grade of late side effects and HRQoL at different time points.

## Patients/material and methods

The present study of PROs was part of the ‘Anal Cancer Radiotherapy – Prospective study of treatment outcome, PROs, utility of imaging and biomarkers, and cancer survivorship (ANCARAD)’, a prospective multi-disciplinary observational trial (NCT1937780). The main inclusion criteria were histologically proven SCCA, planned CRT, and Eastern Cooperative Oncology Group (ECOG) performance status ≤ 2. Patients were included between October 2013 and September 2017 at Oslo University Hospital (OUS), Norway, and a total of 141 patients were included, whereof 132 were eligible for analysis [3]. The ANCARAD study was approved by the Regional Ethical Committee South-East (2012/2274) and the OUS Institutional Study Board. All patients signed an informed consent.

Treatment was given according to national guidelines at that time, after staging according to the tumor-node-metastasis (TNM) classification, 7th Edition [13]. Detailed treatment information is described in the previous publication on outcomes [3]. In short, recommended gross tumor volume dose was 54 Gy for T1–2N0 and 58 Gy for T3–T4 or N1–3 disease. Lymph node metastases received 54 Gy if < 2 cm and 58 Gy if > 2 cm, and the elective clinical target volume dose was 46 Gy. Radiotherapy was given either by Intensity Modulated Radiotherapy (IMRT)/Volumetric Modulated Arc Therapy (VMAT) or conformal 3-D radiotherapy, and either with a sequential boost or a simultaneous integrated boost. Concomitant chemotherapy was delivered with one or two cycles of mitomycin C and 5-fluorouracil/capecitabine.

PROMs were completed pretreatment/at start of treatment (baseline), 3 months after completed CRT, and at 1-, 3-, and 5-year follow-up. Since the study did not involve an experimental intervention, the study protocol had a pragmatic design. The study follow-up and PROMs were aligned with the routine clinical follow-up, resulting in some visits being postponed, for instance during holidays in asymptomatic patients with complete response. Patients completed the questionnaires on paper forms in the outpatient clinic. During the COVID-19 pandemic, some follow-up visits were conducted via phone with PROMs sent by mail. Therefore, a wide time range was accepted for the time points. Baseline included forms from inclusion until day 7 of CRT. At 3 months, we accepted forms from day 25 to 188 after CRT, since some patients were followed for 6 months before final response evaluation. At 1-, 3-, and 5-year follow-up, forms completed roughly between 6 months before and after the actual time point were accepted.

### Patient- reported outcome measures

The EORTC QLQ-C30 is a reliable and valid measure to assess the HRQoL of cancer patients [14]. The EORTC QLQ-CR29 is a validated module specific for colorectal cancer [15], and since there was no anal cancer-specific HRQoL questionnaire at the time this study was conducted, this was used in addition to other questionnaires. For both forms, a score is calculated from 0 to 100. A high score for a functional scale represents a high level of functioning, and a high score for a symptom scale represents a high symptom burden [16]. The differences in score are often reported as small (5–10), moderate (10–20), or large (>20) [17]. Based on this, we regarded a change in score of ≥10 points to be defined as clinically relevant.

St. Marks incontinence score (SMIS) measures fecal incontinence over the last four weeks. It consists of four questions scored from ‘never’ to ‘daily’, and three questions answered with yes or no [18]. SMIS was originally designed to be scored by health personnel, but the use as a self-reporting tool has been validated [19]. Due to administrative failure, baseline assessments were available only for a very small proportion of patients, and baseline results were therefore excluded from analysis.

The Fatigue Questionnaire includes 11 questions to assess the extent of fatigue symptoms last month compared to when they last felt well. Although not specifically validated for cancer patients, it has been widely used in oncology research. Each question is scored from 0 to 3, giving a total score from 0 to 33. Questions 1–7 relate to physical fatigue, and questions 8–11 to mental fatigue [20]. A clinically relevant difference between timepoints has not been included, as no published thresholds for clinically meaningful change in this fatigue questionnaire are currently available. Chronic fatigue (CF) is defined by the duration of symptoms ≥ 6 months and a score of ≥ 4 in a dichotomized (0 = 0, 1 = 0, 2 = 1, 3 = 1) scale [21].

The Hospital Anxiety and Depression Scale (HADS) is a self-reported questionnaire containing two subscales: one for anxiety (HADS-A) and one for depression (HADS-D). Each scale has seven questions, scored from 0 to 3 depending on severity the last week, giving a possible total score of 0–21 for each subscale, and higher scores mean higher burden [22].

A shortened form of the Eysenck Personality Questionnaire was used to detect neuroticism traits among the patients [23]. The form contains six questions, each scored from 0 to 1, giving a maximal score of 6. In line with other studies on cancer patients, we chose to dichotomize the score to low (score 0–2) or high (score 3–6) neuroticism [24–27].

In the figures illustrating clinically relevant and statistically significant changes based on the EORTC QLQ-C30 and EORTC QLQ-CR29, we included mean scores from a control group. This control group consisted of 1693 individuals aged ≥18 years with no history of colorectal cancer. The reference values were obtained from a publication by the Cancer Registry of Norway, which reported PROs among individuals treated with curative intent for rectal cancer [28].

## Statistics

Descriptive statistics are presented using mean and standard deviation or median and range for continuous variables and absolute and relative frequencies for categorical variables.

Linear mixed models were used to assess changes in mean scores over time. This model was chosen because it can handle missing data and utilize all available data, not just data from patients with complete responses. No formal adjustment for multiple testing was performed. Only results with ≥ 10 point change and a *p*-value less than 0.01 were regarded as significant.

To assess potential associations between various covariates and total fatigue, we used multiple linear regression models. Associations with the same covariates and CF were estimated with multiple logistic regression models. Covariates included in the models were selected a priori based on clinical experience and by consulting previous publications.

Missing items for Fatigue Questionnaire and HADS were imputed with the mean value of the other answers in the same questionnaire for that selected time, if at least 50% were answered, according to the ‘half way rule’ [29]. Regarding SMIS, we only included complete cases, since a mean score is difficult to interpret. For EORTC questionnaires, missing items were handled according to the scoring manual [16]. Analyses were performed using SPSS version 25.0.

## Results

Among the 132 patients eligible for analyses in the main study, 12 patients were excluded from PROs analyses due to language difficulties (2), poor compliance (3), and patient preference to not participate with PROMs (7), leaving 120 patients who completed at least one questionnaire and were eligible for analysis. Median age was 62 years (range 39–83), 70.8% were females, 19.1% had a previous or simultaneous other cancer, 41.6% had T3–T4 tumors, and 44.2% had N1–3 disease (Table 1).

**Table 1:** Patient, tumor, and treatment characteristics.

Patient, tumor, and treatment characteristics	*N* (%)
	*n* = 120
**Age**	
Median (range), years	62 (39–83)
**Sex**	
Female	85 (71)
Male	35 (29)
**Current smoking**	
Yes	38 (32)
No	79 (66)
Unknown	3 (3)
**Known HIV-status**	
Positive	3 (3)
**Second cancer diagnosis**	
Previous and/or simultaneous	23 (19)
HPV high risk status*	
Positive	96 (80)
Negative	23 (19)
Unknown	1 (1)
**Organ transplant**	
Yes	1 (1)
**Pre-treatment stoma**	
Yes	9 (8)
No	109 (91)
Stoma placement during treatment	2 (2)
**Tumor localization**	
Anal margin only	11 (9)
Anal canal and/or anal margin and/or rectum	109 (91)
**T-stage**	
T1	15 (13)
T2	55 (46)
T3	22 (18)
T4	28 (23)
**N stage**	
N0	67 (56)
N1	11 (9)
N2	22 (18)
N3	20 (17)
**M stage**	
M0	119 (99)
M1**	1 (1)
**Radiotherapy technique**	
Conventional RT	37 (31)
IMRT	10 (8)
VMAT	73 (61)
**Radiotherapy boost**	
Sequential boost	107 (89)
Simultaneous integrated boost	13 (11)
Concomitant chemotherapy***	
MMC + 5FU – one cycle	56 (47)
MMC + 5FU – two cycles	53 (44)
Other	5 (4)
No chemotherapy	6 (5)

* HPV 16, 18, 31, 33, 35, 45, 52, 56, 58, or 66.

** Lymph node metastases (paraaortic) included in radiation field.

*** MMC + capecitabine (*n* = 1). MMC omitted in second cycle (*n* = 5).

Other: 5-FU monotherapy (*n* = 1), two cycles of cisplatin + 5-FU (*n* = 1) and neoadjuvant and concomitant Cisplatin + 5-FU and/or MMC + 5-FU (*n* = 3).

### EORTC QLQ-C30

The mean scores at the different time points are presented in Supplementary Table 1A. Global QoL, role functioning, emotional function, dyspnea, and appetite loss showed a statistically significant linear trend over time (*p* < 0.01).

From baseline to 3 months after CRT, constipation changed for the better at a clinically relevant and statistically significant level (difference 10.1, *p* < 0.001). From 3 months to 5 years, role functioning (difference 11.2, *p* < 0.001), appetite loss (difference 11.9, *p* < 0.001), and diarrhea (difference 10, *p* < 0.005) changed for the better at a clinically relevant and statistically significant level (Table 2A).

**Table 2A:** Mean scores and change in score between baseline and 3 months, and 3 months and 5 years, after chemoradiotherapy.

	Baseline	95% CI	Change from baseline to 3 months			Change from 3 months to 5 years		
			3 months	95% CI	*p*	5 years	95% CI	*p*
**A) EORTC QLQ-C30**								
Global QoL	63.3	58.5–68.1	67.1	62.4 -71.7	0.113	72	67.1–76.9	0.057
Physical functioning	80.4	76.7–84.1	74.8	71.3–78.3	**0.002**	75.5	71.8–79.2	0.725
Role functioning	68.9	62.7–75.1	64.1	58–70.2	0.115	75.3	68.9–81.7	< 0.001*
Emotional functioning	75.5	71.7–79.3	75.6	72.1–79.1	0.535	83.1	79.4–86.8	**< 0.001**
Cognitive functioning	80	75.4–84.6	78.6	74.5–82.7	0.502	79	74.6–83.4	0.461
Social functioning	73	67.8–78.3	66.6	60.7–72.5	0.032	72.6	66.3–78.9	0.242
Fatigue	36.3	30.9–41.6	38.5	33.7–41.1	0.365	33	27.9–38.1	0.041
Nausea and vomiting	5.8	3.6–8	6.7	4–9.4	0.499	6.2	3.4–9	0.743
Pain	32.4	26.4–38.6	24.8	26.3–30.9	0.015	27.3	20.8–33.8	0.469
Dyspnea	13.9	10–17.8	19.9	15.1–24.7	0.014	21.1	16–26.2	0.677
Insomnia	37.6	31.3–43.9	33	26.7–39.3	0.151	34.6	27.9–41.3	0.650
Appetite loss	23.6	17.4–29.8	19.6	13.9–25.3	0.171	7.7	1.7–13.7	< 0.001*
Constipation	26.3	20–32.6	16.2	10.2–22.2	< 0.001*	22.1	15.7–28.5	0.081
Diarrhea	25.7	20.9–30.5	32.6	26.2–39	0.034	22.6	15.8–29.4	0.005*
Financial difficulties	5.8	2.7–8.9	10.1	6–14.2	0.037	7.7	3.4–12	0.295

P values in bold are statistically significant (p < 0.01), and if marked with * the change is both clinically relevant (change ≥ 10) and statistically significant (p < 0.01).

**Figure 1:**
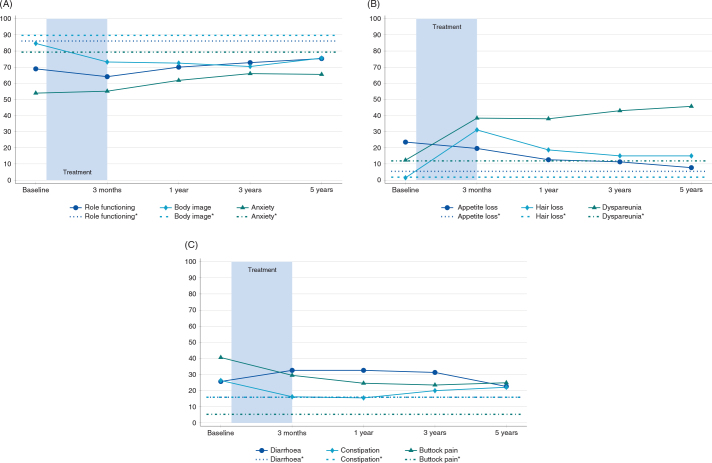
EORTC-QLQ-C30 and EORTC-QLQ-CR29. Mean values at inclusion, 3 months, 1 year, 3 years, and 5 years after end of chemoradiotherapy. *The reference population includes individuals aged 18 and above, living in Norway, who have not previously been diagnosed with colorectal cancer. Ref: Slørdahl KS et al.^28^ (A) Functional scales; Body image deteriorated from baseline to 3 months, while role functioning and anxiety improved from 3 months to 5 years. (B) Symptom scales; Dyspareunia and hair loss worsened from baseline to 3 months, and appetite loss improved from 3 months to 5 years after treatment. (C) Symptom scales; Constipation and buttock pain improved from baseline to 3 months, and diarrhea improved from 3 months to 5 years after treatment.

Figure 1 shows functional- and symptom scales with clinically relevant and statistically significant changes, with the scores from a reference population included for comparison.

### EORCT-QLQ-CR29

The mean scores at different time points are presented in Supplementary Table 1B. Body image, anxiety, urinary incontinence, buttock pain, impotence, dyspareunia, and flatulence had a statistically significant linear trend (*p* < 0.01).

From baseline to 3 months after CRT, we found a statistically significant and clinically relevant change for the worse for body image (difference 11.5, *p* < 0.001), hair loss (difference 29.7, *p* < 0.001), and dyspareunia (difference 26, *p* < 0.001), whereas buttock pain changed for the better (difference 11.1, *p* = 0.002). From 3 months to 5 years, there was a statistically significant and clinically relevant change for the better regarding anxiety (difference 10.4, *p* < 0.001) and hair loss (difference 16.1, *p* < 0.001) (Table 2B).

**Table 2B:** Mean scores and change in score between baseline and 3 months, and 3 months and 5 years, after chemoradiotherapy.

	Baseline	95% CI	Change from baseline to 3 months			Change from 3 months to 5 years		
			3 months	95% CI	*p*	5 years	95% CI	*p*
**B) EORTC QLQ-CR29**								
Body image	84.7	80.8–88.7	73.2	68–78.4	< 0.001*	75.6	70.1–81.1	0.402
Anxiety	53.9	49.1–58.8	55.1	49.4–60.8	0.670	65.5	59.4–71.6	0.001*
Weight	82.2	77.5–87	82.6	76.9–88.3	0.880	86.6	80.6–92.6	0.191
Sexual interest (male)	37.5	27.5–47.5	36.7	27.2–46.2	0.804	40.1	30.1–50.2	0.399
Sexual interest (female)	14.7	9.2–20.2	12.4	6.9–17.9	0.412	17.5	11.7–23.3	0.086
Urinary frequency	26	21.1–30.9	24.1	18.6–29.6	0.484	29.8	24–35.6	0.044
Urinary incontinence	5.9	2.9–8.9	6.1	2.2–10	0.939	12.2	8–16.4	**0.004**
Dysuria	3.5	1.1–5.9	8	4.9–11.1	**0.005**	4.3	1–7.6	0.032
Abdominal pain	12.5	8.1–16.9	16.7	11.8–21.6	0.087	15.3	10.1–20.5	0.592
Buttock pain	40.6	33.5–47.7	29.5	22.4–36.6	0.002*	24.9	17.4–32.4	0.237
Bloating	21.2	16.1–26.3	22.7	17.5–27.9	0.554	23.2	17.7–28.7	0.873
BMS	20.9	16.4–25.4	13.9	9.1–18.7	**0.004**	14.7	9.6–19.8	0.768
Dry mouth	22.4	16.8–28	26.5	21–32	0.153	24.8	18.9–30.7	0.606
Hair loss	1.4	0.04–2.8	31.1	24.5–37.7	< 0.001*	15	5–8	< 0.001*
Taste	8.2	4.2-12.2	18.1	13.5–22.7	**<0.001**	11.7	6.9–16.5	0.010
Impotence	44.1	30.6–57.6	46.1	34–58.2	0.739	56.9	43.7–70.1	0.073
Dyspareunia	12.4	5.5–19.3	38.4	27.3–49.5	< 0.001*	45.7	34.2–57.2	0.204
Flatulence	28.5	22.4–34.6	37.6	31–44.2	**0.007**	45.9	38.9–52.9	0.026
Fecal incontinence	16.9	11.8–22	24.2	17.8–30.6	0.024	27.8	21–34.6	0.344
Sore skin	34.5	27.8–41.2	29.9	22.3–37.5	0.236	28.4	20.4–36.4	0.763
Stool frequency	12	8.4–15.6	19.4	14.9–23.9	**0.001**	16	11.2–20.8	0.180
Embarrassment	7.1	3.4–10.8	8	2.2–13.8	0.755	10.7	4.6–16.8	0.408

P values in bold are statistically significant (p < 0.01), and if marked with * the change is both clinically relevant (change ≥ 10) and statistically significant (p < 0.01).

Figure 1 shows functional- and symptom scales with clinically significant relevant change, compared to a reference population.

### St. Marks score

Mean score at 5 years was 8.8 compared to 8.9 at 3 months. The number of patients with incontinence problems of different degrees was relatively stable from 3 months to 5 years. As many as 60.3% of the patients experienced the lack of ability to defer defecation for 15 min at 5 years, and 23.5% of the patients needed to wear a pad or plug at 5 years due to fecal incontinence (Supplementary Figure 1).

### Fatigue

The mean total fatigue score was 14.6 pretreatment, rising to 17.1 at 3 months before declining to 15.5 at 5 years. The change in score was significantly different from baseline to 3 months and from 3 months to 5 years (Table 3). The change from baseline to 5 years was not statistically significant (*p* = 0.073). Scores at all time points is shown in Supplementary Table 2. Previous or simultaneous cancer at diagnosis was associated with higher total fatigue at baseline (coefficient 2.4, confidence interval (CI) 0.04 – 4.7, *p* = 0.046) and at 5 years (coefficient 3.4, CI 0.1 – 6.7, *p* = 0.042), but this factor did not remain significant in a model adjusting for age, gender, neuroticism, and high risk based on N- and T-stage. Age ≥ 70 years was associated with a lower fatigue score at 3 months, 3 years, and 5 years, most pronounced at 5 years (coefficient –4.8, CI –8.2 to –1.3, *p* = 0.008), whereas neuroticism was associated with a higher fatigue score at all time points, most pronounced at 3 years (coefficient 3.7, CI 0.8 – 6.6, *p* = 0.013) (Supplementary Table 3).

**Table 3:** Fatigue score at baseline, 3 months, and 5 years after chemoradiotherapy.

Fatigue	Baseline		3 months		Change from baseline to 3 months	5 years		Change from 3 months to 5 years
	Score	95% CI	Score	95% CI	*p*	Score	95% CI	*p*
Total fatigue	14.6	13.7–15.5	17.1	15.2–18.9	< 0.001	15.5	13.6–17.4	0.003
Physical fatigue	9.9	9.2–10.6	11.9	10.4–13.3	< 0.001	10.4	8.9 –11.9	< 0.001
Mental fatigue	4.8	4.5–5.0	5.3	4.7–5.9	0.006	5.2	4.6–5.8	0.694

CF was present among 20 patients (18%) at baseline, declining to 15 patients (16%) at 3 months, before rising to 39 patients (41%) at 1 year, 35 patients (37%) at 3 years, and 23 patients (28%) at 5 years (Table 4). Age ≥ 70 years was associated with an increased risk for CF (odds ratio (OR) 9, CI 1.8 – 45.3, *p* = 0.008 in multivariable analysis) at 1 year (Supplementary Table 4). The presence of CF was associated with reduced global health status/QoL. At 5 years, patients with CF had a global health status/QoL of 58 vs 80.9 among patients without CF.

**Table 4:** Chronic fatigue at baseline, 3 months, 1 year, 3 years, and 5 years after chemoradiotherapy.

Presence of chronic fatigue	Baseline		3 months		1 year		3 years		5 years	
	*n* = 110		*n* = 97		*n* = 95		*n* = 94		*n* = 82	
	*n*	%	*n*	%	*n*	%	*n*	%	*N*	%
Yes	20	18.2	15	15.5	39	41.1	35	37.2	23	28.1
No	66	60	66	68	40	42.1	40	42.6	38	46.3
Unknown	24	21.8	16	16.5	16	16.8	19	20.2	21	25.6

### Hospital anxiety and depression scale

Mean HADS-A score at baseline was 4.8, rising to 5.0 at 3 months (*p* = 0.645) before declining to a mean score of 4.2 at 5 years (*p* = 0.032). Regarding HADS-D, the mean score at baseline was 3.0, before increasing to 3.7 at 3 months (*p* = 0.023), and before declining to a level of 3.3 at 5 years (*p* = 0.154) (Supplementary Table 5).

### Eysenck personality questionnaire

A total of 25 patients (22.3%) who answered the questionnaire had a high score for neuroticism.

## Discussion and conclusion

This study reveals that patients treated with CRT have a high symptom burden before start of treatment, presumably due to the cancer disease itself. Three months after CRT, some symptoms improve, while an exacerbation of several symptoms and functional outcomes is observed. Some functional outcomes improve with time, but most domains remain impaired, especially for anorectal and female sexual function, where the symptom burden remains high. Approximately one-third of patients report to have CF at 5 years after CRT.

The presence of a tumor in the anal canal may lead to local symptoms, such as buttock pain, constipation, and blood/mucus in the stool [30] and is likely the cause of the high symptom levels for these domains before treatment. We did not measure PROs at the end of treatment, but in the ACT4 study, it was evident that acute toxicity is worse at that time point, and that most has resolved by 3 months [31]. This study shows that these symptoms, in particular buttock pain, already are significantly improved 3 months after CRT, compatible with tumor response resulting in less symptoms from the tumor itself [32]. This is in line with Gilbert et al. and Lefèvre et al. findings [11, 33]. Compared to normative data [28, 34], the pretreatment scores of many domains in this study are worse, which may express that the cancer disease itself, in addition to the local symptoms, influences HRQoL. Anorectal, sexual, and urinary symptoms were found to become worse three months after treatment and to remain impaired 5 years after treatment. This is in line with recent results from the Swedish national study [35] and may be explained by the known late side effects after radiation therapy (RT) against the pelvic region. Around 30% of patients in the current study received 3D conformal RT, and the majority received IMRT/VMAT; the impact of the techniques on late toxicity is not fully known. Social functioning scores persist low and may possibly be associated with anorectal dysfunction [36]. Gilbert and Lefèvre et al. [11, 33] described an improvement from before start of treatment to 1 year, which this study only found for some of the functional outcomes and the symptoms mentioned earlier.

CF was found among 28% at 5 years follow-up, with the highest level at 1 year of 41%. To our knowledge, this is the first time the rate of CF is reported in anal cancer survivors. CF has been reported in survivors of breast cancer (33–39%) [37], Hodgkins lymphoma (42%) [38], and head and neck cancer (33%) [39]. There is still a lack of understanding for the mechanisms causing CF. Except for age ≥70 years at 1 year, increasing the risk for CF, we did not find any significant predictors for CF. This result should also be interpreted with caution due to low number of patients in each group. More research should be conducted for potential treatment-related risk factors for CF among anal cancer survivors, including systemic treatment and RT (field size, technique, CRT, etc.)-related factors. Moreover, the precise etiological factors remain unidentified, and further research, including the search for potential biomarkers, is necessary. Although not validated specifically for cancer patients, the Fatigue Questionnaire appears to be a useful tool for assessing fatigue, as it contains 11 items capturing both physical and mental dimensions, such as concentration and memory. Fatigue is also measured with the EORTC QLQ-C30; however, the score only consists of three items related to the need for rest, feeling weak or being tired, and it may not fully capture all aspects.

Despite high survival rates [3], this study confirms that patients treated for anal cancer suffer from late side effects and reduced HRQoL after CRT. The goal for future treatment is to maintain or improve the survival rates and, at the same time, deliver the optimal personalized treatment and avoid unnecessary late side effects. The Nordic Anal Cancer Group recently reviewed the evidence for clinical target volume contouring and suggested risk-adapted target volumes [40]. There are ongoing clinical trials aiming to personalize and determine the optimal RT dose (ACT 3, 4, 5 (ISRCTN88455282)) [31, 41] and (EA2182 DECREASE (NCT04166318)). The ACT4 has recently shown that reduced dose to patients with T1–2(≤ 4 cm)N0M0 anal cancer is well tolerated with maintained early oncological outcomes [31]. Other studies aim to determine if there is a role for proton therapy (SWANCA (NCT04462042)) in the treatment of anal cancer. The possible effect of immunotherapy is also being investigated (CORINTH (NCT04046133) and EA2165 trial (NCT03233711)) [42]. A more tailored treatment regimen will hopefully improve outcomes and reduce late side effects; however, some will likely persist, such as anorectal late side effects due to the localization of the tumor and the radiation doses needed for cure. It is important to continue to investigate PROs if treatment recommendations change to capture potential changes in late side effects.

The management of late side effects is important, and up to recent years, the recommendations for this patient group have been insufficient [43]. Norwegian guidelines for management of late side effects after cancer treatment, dedicates one chapter to management of various late side effects after treatment for pelvic cancer [44]. European Society For Medical Oncology (ESMO) guidelines suggests patient and family education, physical activity, and psychosocial intervention as the possible management skills of cancer-related fatigue [45]. Hormone replacement therapy and vaginal dilatation for sexual dysfunction, or antidiarrheal drugs and sacral nerve stimulation for fecal incontinence, are some of many recommendations published by Haas et al. for the management of late side effects; however, many have evidence level D [46]. Furthermore, patients living with side effects often develop coping strategies and undergo a response shift. Although there is more attention directed to the management of late side effects and HRQoL, there is still lack of evidence of the best management, and more studies should be encouraged.

The strength of this study is the large number of patients and the prospective design with data from several time points. Limitations are the lack of an anal cancer-specific questionnaire and more detailed information regarding sexual function and potential late side effects regarding the pelvic bones, such as osteoporosis or pelvic bone insufficiency fractures [47]. There was variation in the collection times of the PROMs; however, these seem to be relatively stable in the long-term follow-up. For the sake of complete variation with time, it is a limitation that we did not measure the worst acute toxicity at end of treatment. Furthermore, there was no information about potential comorbidities contracted during the follow-up period, possibly affecting HRQoL. Although the linear mixed model was considered the most appropriate method, there may be a possibility of systematic dropout.

In conclusion, patients experience a high symptom burden and reduced HRQoL before start of treatment. Many disease-specific symptoms have improved by 3 months; however, anorectal and sexual dysfunction remain. CF is observed 5 years after CRT, in particular, in older patients. These findings demonstrate the prolonged impact of CRT on patients’ quality of life, emphasizing the need for ongoing surveillance and the management of late effects during follow-up to optimize patient care.

## Supplementary Material



## Data Availability

The data that support the findings of this study are not publicly available due to privacy. Data can be made available upon reasonable request to the corresponding author, subject to appropriate ethical approval.

## References

[1] Rao S, Guren MG, Khan K, Brown G, Renehan AG, Steigen SE, et al. Anal cancer: ESMO clinical practice guidelines for diagnosis, treatment and follow-up(*). Ann Oncol. 2021;32(9):1087–100. 10.1016/j.annonc.2021.06.015 34175386

[2] Sekhar H, Malcomson L, Kochhar R, Sperrin M, Alam N, Chakrbarty B, et al. Temporal improvements in loco-regional failure and survival in patients with anal cancer treated with chemo-radiotherapy: treatment cohort study (1990–2014). Br J Cancer. 2020;122(6):749–58. 10.1038/s41416-019-0689-x 31932755 PMC7078229

[3] Slørdahl KS, Klotz D, Olsen J, Skovlund E, Undseth C, Abildgaard HL, et al. Treatment outcomes and prognostic factors after chemoradiotherapy for anal cancer. Acta Oncol. 2021;60(7):921–30. 10.1080/0284186X.2021.1918763 33966592

[4] Besson A, Deftereos I, Chan S, Faragher IG, Kinsella R, Yeung JM. Understanding patient-reported outcome measures in colorectal cancer. Future Oncol. 2019;15(10):1135–46. 10.2217/fon-2018-0723 30880455

[5] Di Maio M, Basch E, Denis F, Fallowfield LJ, Ganz PA, Howell D, et al. The role of patient-reported outcome measures in the continuum of cancer clinical care: ESMO clinical practice guideline. Ann Oncol. 2022;33(9):878–92. 10.1016/j.annonc.2022.04.007 35462007

[6] Fish R, Sanders C, Adams R, Brewer J, Brookes ST, DeNardo J, et al. A core outcome set for clinical trials of chemoradiotherapy interventions for anal cancer (CORMAC): a patient and health-care professional consensus. Lancet Gastroenterol Hepatol. 2018;3(12):865–73. 10.1016/S2468-1253(18)30264-4 30507470

[7] Samuel R, Knight SR, Adams R, Das P, Dorth J, Finch D, et al. International consensus to define outcomes for trials of chemoradiotherapy for anal cancer (CORMAC-2): defining the outcomes from the CORMAC core outcome set. EClinicalMedicine. 2024;78:102939. 10.1016/j.eclinm.2024.102939 39720423 PMC11667046

[8] Pe M, Voltz-Girolt C, Bell J, Bhatnagar V, Bogaerts J, Booth C, et al. Using patient-reported outcomes and health-related quality of life data in regulatory decisions on cancer treatment: highlights from an EMA-EORTC workshop. Lancet Oncol. 2025;26(6):687–90. 10.1016/S1470-2045(25)00150-0 40245904

[9] Bentzen AG, Balteskard L, Wanderås EH, Frykholm G, Wilsgaard T, Dahl O, et al. Impaired health-related quality of life after chemoradiotherapy for anal cancer: late effects in a national cohort of 128 survivors. Acta Oncol. 2013;52(4):736–44. 10.3109/0284186X.2013.770599 23438358

[10] Sodergren SC, Vassiliou V, Dennis K, Tomaszewski KA, Gilbert A, Glynne-Jones R, et al. Systematic review of the quality of life issues associated with anal cancer and its treatment with radiochemotherapy. Support Care Cancer. 2015;23(12):3613–23. 10.1007/s00520-015-2879-2 26289529

[11] Lefèvre AC, Serup-Hansen E, Storm KS, Wind KL, Kronborg C, Spindler KG. One-year treatment-related side effects and quality of life after chemoradiotherapy in squamous cell carcinoma of the anus. Int J Radiat Oncol Biol Phys. 2023;115(5):1165–77. 10.1016/j.ijrobp.2022.09.071 36179989

[12] Cancer registry of Norway. Cancer Registry of Norway, Norwegian Institute of Public Health. Cancer in Norway 2022 – cancer incidence, mortality, survival and prevalence in Norway. Oslo: Cancer Registry of Norway; 2023. Oslo; 2022.

[13] Sobin LH, editor., MKGE, Wittekind C. (Editor). TNM classifiaction of malignant tumours. Oxford, Wiley. 7th ed. 2011.

[14] Aaronson NK, Ahmedzai S, Bergman B, Bullinger M, Cull A, Duez NJ, et al. The European Organization for Research and Treatment of Cancer QLQ-C30: a quality-of-life instrument for use in international clinical trials in oncology. J Natl Cancer Inst. 1993;85(5):365–76. 10.1093/jnci/85.5.365 8433390

[15] Whistance RN, Conroy T, Chie W, Costantini A, Sezer O, Koller M, et al. Clinical and psychometric validation of the EORTC QLQ-CR29 questionnaire module to assess health-related quality of life in patients with colorectal cancer. Eur J Cancer. 2009;45(17):3017–26. 10.1016/j.ejca.2009.08.014 19765978

[16] Fayers PM AN, Bjordal K, Groenvold M, Curran D, Bottomley A, on Group. botEQoL. The EORTC QLQ-C30 scoring manual. Brussel, European Organisation for Research and Treatment of Cancer. 3rd ed. 2001.

[17] Osoba D, Rodrigues G, Myles J, Zee B, Pater J. Interpreting the significance of changes in health-related quality-of-life scores. J Clin Oncol. 1998;16(1):139–44. 10.1200/JCO.1998.16.1.139 9440735

[18] Vaizey CJ, Carapeti E, Cahill JA, Kamm MA. Prospective comparison of faecal incontinence grading systems. Gut. 1999;44(1):77–80. 10.1136/gut.44.1.77 9862829 PMC1760067

[19] Johannessen HH, Norderval S, Stordahl A, Falk RS, Wibe A. Interview-based versus self-reported anal incontinence using St Mark’s incontinence score. Int Urogynecol J. 2018;29(5):667–71. 10.1007/s00192-017-3363-y 28547271

[20] Chalder T, Berelowitz G, Pawlikowska T, Watts L, Wessely S, Wright D, et al. Development of a fatigue scale. J Psychosom Res. 1993;37(2):147–53. 10.1016/0022-3999(93)90081-P 8463991

[21] Dahl AA, Grotmol KS, Hjermstad MJ, Kiserud CE, Loge JH. Norwegian reference data on the fatigue questionnaire and the Patient Health Questionnaire-9 and their interrelationship. Ann Gen Psychiatry. 2020;19:60. 10.1186/s12991-020-00311-5 33062033 PMC7547512

[22] Zigmond AS, Snaith RP. The hospital anxiety and depression scale. Acta Psychiatr Scand. 1983;67(6):361–70. 10.1111/j.1600-0447.1983.tb09716.x 6880820

[23] Eyseneck SBG, Tambs K. Cross-cultural comparison of personality: Norway and England. Scand J Psychol. 1990;31:191–7. 10.1111/j.1467-9450.1990.tb00830.x

[24] Bøhn SH, Vandraas KF, Kiserud CE, Dahl AA, Thorsen L, Ewertz M, et al. Work status changes and associated factors in a nationwide sample of Norwegian long-term breast cancer survivors. J Cancer Surviv. 2024;18(2):375–84. 10.1007/s11764-022-01202-2 35314959 PMC10960762

[25] Dahl AA, Smedsland SK, Vandraas KF, Bøhn SK, Falk RS, Kiserud CE, et al. High neuroticism is associated with common late adverse effects in a nationwide sample of long-term breast cancer survivors. Breast Cancer Res Treat. 2023;202(1):97–104. 10.1007/s10549-023-07055-2 37528264 PMC10504095

[26] Grav S, Stordal E, Romild UK, Hellzen O. The relationship among neuroticism, extraversion, and depression in the HUNT study: in relation to age and gender. Issues Ment Health Nurs. 2012;33(11):777–85. 10.3109/01612840.2012.713082 23146012

[27] Grov EK, Dahl AA. Is neuroticism relevant for old cancer survivors? A controlled, population-based study (the Norwegian HUNT-3 survey). Support Care Cancer. 2021;29(7):3623–32. 10.1007/s00520-020-05870-7 33174077 PMC8163664

[28] Slørdahl KS, Balto A, Guren MG, Wibe A, Kørner H, Norderval S, et al. Patient-reported outcomes after treatment for rectal cancer – a prospective nationwide study. Colorectal Dis. 2024;27(1):e17231. 10.1111/codi.17231 39515999 PMC11683195

[29] Bell ML, Fairclough DL, Fiero MH, Butow PN. Handling missing items in the Hospital Anxiety and Depression Scale (HADS): a simulation study. BMC Res Notes. 2016;9(1):479. 10.1186/s13104-016-2284-z 27770833 PMC5075158

[30] Helsedirektoratet. Analkreft – handlingsprogram [Internet]. Helsedirektoratet; 2021. Available from: https://www.helsedirektoratet.no/retningslinjer/analkreft-handlingsprogram [Cited date: 02.07.24]

[31] Gilbert A, Adams R, Webster J, Gilbert DC, Abbott NL, Berkman L, et al. Standard versus reduced-dose chemoradiotherapy in anal cancer (PLATO-ACT4): short-term results of a phase 2 randomised controlled trial. Lancet Oncol. 2025;26(6):707–18. 10.1016/S1470-2045(25)00213-X 40334666

[32] Caravatta L, Mantello G, Valvo F, Franco P, Gasparini L, Rosa C, et al. Radiotherapy with intensity-modulated (IMRT) techniques in the treatment of anal carcinoma (RAINSTORM): a multicenter study on behalf of AIRO (Italian Association of Radiotherapy and Clinical Oncology) Gastrointestinal Study Group. Cancers (Basel). 2021;13(8):1902. 10.3390/cancers13081902 33920873 PMC8071256

[33] Gilbert A, Drinkwater K, McParland L, Adams R, Glynne-Jones R, Harrison M, et al. UK national cohort of anal cancer treated with intensity-modulated radiotherapy: one-year oncological and patient-reported outcomes. Eur J Cancer. 2020;128:7–16. 10.1016/j.ejca.2019.12.022 32109852

[34] Åsberg RE, Nilsen M, Hjermstad MJ, Reinertsen KV, Karlsen J, Giskeødegård GF, et al. Norwegian general population normative data for the European Organization for Research and Treatment of Cancer questionnaires: the Quality of Life Questionnaire-Core 30, the Sexual Health Questionnaire QLQ-SHQ22 and the sexual domains of the QLQ-BR23/BR45. Eur J Cancer. 2023;190:112943. 10.1016/j.ejca.2023.112943 37515905

[35] Axelsson A, Johansson M, Haglind E, Li Y, Nilsson PJ, Angenete E. Patient reported long-term side effects from treatment on urinary and sexual function in anal cancer survivors – 3- and 6-year results from the Swedish national ANCA study. Colorectal Dis. 2024;26(7):1359–69. 10.1111/codi.17040 38816903

[36] Bentzen AG, Guren MG, Vonen B, Wanderås EH, Frykholm G, Wilsgaard T, et al. Faecal incontinence after chemoradiotherapy in anal cancer survivors: long-term results of a national cohort. Radiother Oncol. 2013;108(1):55–60. 10.1016/j.radonc.2013.05.037 23891095

[37] Reinertsen KV, Cvancarova M, Loge JH, Edvardsen H, Wist E, Fosså SD. Predictors and course of chronic fatigue in long-term breast cancer survivors. J Cancer Surviv. 2010;4(4):405–14. 10.1007/s11764-010-0145-7 20862614 PMC2978315

[38] Eikeland SA, Smeland KB, Simensen VC, Fagerli UM, Bersvendsen HS, Kiserud CE, et al. Chronic fatigue in long-term survivors of Hodgkin’s lymphoma after contemporary risk-adapted treatment. Acta Oncol. 2023;62(1):80–8. 10.1080/0284186X.2023.2168215 36715320

[39] Huynh TM, Falk RS, Hellebust TP, Dale E, Astrup GL, Hjermstad MJ, et al. Chronic fatigue in long-term survivors of head and neck cancer treated with radiotherapy. Radiother Oncol. 2024;195:110231. 10.1016/j.radonc.2024.110231 38518958

[40] Nilsson MP, Undseth C, Albertsson P, Eidem M, Havelund BM, Johannsson J, et al. Nordic anal cancer (NOAC) group consensus guidelines for risk-adapted delineation of the elective clinical target volume in anal cancer. Acta Oncol. 2023;62(8):897–906. 10.1080/0284186X.2023.2240490 37504978

[41] ISRCTN. PLATO trial: personalising anal cancer radioTherapy dOse – incorporating ACT3, ACT4 and ACT5 2016: ISRCTN88455282. 10.1186/ISRCTN88455282

[42] Guren MG, Sebag-Montefiore D, Franco P, Johnsson A, Segelov E, Deutsch E, et al. Treatment of squamous cell carcinoma of the anus, unresolved areas and future perspectives for research: perspectives of research needs in anal cancer. Clin Colorectal Cancer. 2021;20(4):279–87. 10.1016/j.clcc.2021.09.006 34645589

[43] Wiltink LM, White K, King MT, Rutherford C. Systematic review of clinical practice guidelines for colorectal and anal cancer: the extent of recommendations for managing long-term symptoms and functional impairments. Support Care Cancer. 2020;28(6):2523–32. 10.1007/s00520-020-05301-7 32025805 PMC7181546

[44] Helsedirektoratet. Seneffekter etter kreftbehandling. 2020. Oslo, Helsedirektoratet. Contract No.: ISBN 978-82-8081-610-8.

[45] Fabi A, Bhargava R, Fatigoni S, Guglielmo M, Horneber M, Roila F, et al. Cancer-related fatigue: ESMO Clinical Practice Guidelines for diagnosis and treatment. Ann Oncol. 2020;31(6):713–23. 10.1016/j.annonc.2020.02.016 32173483

[46] Haas S, Mikkelsen AH, Kronborg C, Oggesen BT, Faaborg PM, -Serup-Hansen E, et al. Management of late adverse effects after chemoradiation for anal cancer. Acta Oncol. 2021;60(12):1688–701. 10.1080/0284186X.2021.1983208 34845967

[47] Kronborg CJ, Pedersen BG, Klemmensen J, Lefévre AC, Wind KL, Spindler KG. Pelvic insufficiency fractures and bone pain after radiation therapy for anal cancer: relation to pelvic bone dose-volume parameters. Adv Radiat Oncol. 2023;8(1):101110. 10.1016/j.adro.2022.101110 36483064 PMC9723308

