# Supplementary material for: Patient-reported outcomes after chemoradiotherapy for anal cancer
Source: Acta Oncol. 2025 Jul 30;64:43636. doi: 10.2340/1651-226X.2025.43636 (PMC12320145; doi:10.2340/1651-226X.2025.43636)
Supplement: Supplementary file 1 [file AO-64-43636-s1.pdf]

Supplementary material has been published as submitted. It has not been copyedited, or typeset by Acta Oncologica

Supplementary table 1

A) EORTC-C30. Mean scores, number, and standard deviation (SD) for each scale or item at baseline, and 3 months, 1 year, 3 years and 5 years after chemoradiotherapy.

	Baseline mean	n	SD	3 months mean	n	SD	1 year mean	n	SD	3 years mean	n	SD	5 years mean	n	SD
Global QoL	63.3	110	25.6	68.2	95	21.8	68.9	98	24.7	71.0	95	23.1	74.6	80	20.7
Physical functioning	80.4	110	19.5	75.6	95	20.8	79.8	98	19.4	77.4	96	21.1	79.1	81	19.7
Role functioning	68.9	110	32.7	65.4	95	32	71.8	98	28.4	75.5	96	26.3	79.4	81	26.0
Emotional functioning	75.5	110	20.0	75.8	95	22.6	77.0	98	22.9	80.6	95	19.6	84.2	80	17.5
Cognitive functioning	80	110	24.2	78.4	95	25.5	79.4	98	23.1	77.0	95	24.7	80.6	80	22.7
Social functioning	73	110	27.7	67.0	95	29.1	71.3	98	28.4	71.6	95	28.1	74.8	80	26.1
Fatigue	36.3	110	28.4	38	95	27.8	34.2	98	23.1	36.6	96	23.9	29.7	81	20.4
Nausea and vomiting	5.8	110	11.6	6.7	95	15.5	5.8	98	13.1	4.5	96	9.5	4.9	81	11.3
Pain	32.4	110	32.8	24.6	95	31.7	25.5	98	28.8	28.8	96	29.4	24.5	81	27.9
Dyspnoe	13.9	110	20.9	19.1	94	24.7	18.0	98	24.9	23.3	96	27.0	18.9	81	27.4
Insomnia	37.6	110	33.5	33.7	95	31.7	34.4	98	31.2	30.9	95	29.3	34.2	81	31.6
Appetite loss	23.6	109	32.5	19.6	95	29.8	10.9	98	23.8	8.7	96	20.7	5.3	81	17.0
Constipation	26.3	109	33.4	16.1	95	27	15.6	98	25.9	19.3	95	24.6	19.6	80	27.4
Diarrhoea	25.7	109	25.5	32.3	95	31.3	31.9	96	30.9	30.2	95	29.6	21.1	79	25.1
Financial difficulties	5.8	110	16.2	10.2	95	23.4	12.2	98	24.6	10.5	95	24.4	5.4	80	16.3

B) EORTC-CR29. Mean scores, number, and standard deviation (SD) for each scale or item at baseline, and 3 months, 1 year, 3 years and 5 years after chemoradiotherapy.

	**Baseline mean**	**n**	**SD**	**3 months mean**	**n**	**SD**	**1 year mean**	**n**	**SD**	**3 years mean**	**n**	**SD**	**5 years mean**	**n**	**SD**
Body image	84.7	104	20.3	73.7	95	26.3	72.8	91	28.1	71.3	91	27.7	77.5	81	26.2
Anxiety	53.9	107	25.3	55.8	95	30.5	61.9	91	27.5	66.3	91	27.0	66.7	80	23.1
Weight	82.2	107	24.8	83.2	95	26.6	83.5	91	24.5	83.2	91	27.4	89.3	81	18.9
Sexual interest (male)	37.5	32	27.8	33.3	33	27.6	40.2	29	27.3	43.0	31	27.5	39.1	29	28.3
Sexual interest (female)	14.7	75	24.0	13.9	72	19.2	16.7	66	25.7	15.3	63	20.6	19.4	60	25.5
Urinary frequency	26.0	107	25.7	23.2	95	26.2	22.2	91	26.9	27.1	91	24.6	27.6	81	27.0
Urinary incontinence	5.9	107	15.7	5.6	95	13.5	12.8	91	22.1	13.3	90	20.5	10.7	81	18.9
Dysuria	3.5	105	12.2	7.0	95	18.8	5.5	91	15.9	4.8	91	15.4	4.1	81	17.0
Abdominal pain	12.5	107	22.7	15.8	95	24.2	16.7	90	25.1	14.7	91	24.4	12.3	81	22.0
Buttock pain	40.6	106	36.8	28.7	94	29.2	23.8	91	29.1	22.3	91	29.4	20.4	80	30.2
Bloating	21.2	107	26.5	22.3	94	25.1	25.3	91	26.9	24.5	91	24.3	23.9	81	27.5
BMS	20.9	107	23.7	13.7	95	18.0	15.9	91	20.0	14.5	91	19.4	14.4	81	19.1
Dry mouth	22.4	107	29.2	26.0	95	30.4	20.7	90	26.2	24.5	91	27.1	22.2	81	27.4
Hair loss	1.4	94	6.8	31.6	95	31.6	19.6	90	29.1	15.8	91	26.9	15.0	80	27.0
Taste	8.2	102	20.1	19.3	95	26.9	14.7	91	24.9	11.7	91	20.7	10.7	81	24.1
Impotence	44.1	31	36.9	40.5	28	38.9	59.0	26	36.9	65.3	25	39.1	48.5	22	43.3
Dyspareunia	12.3	54	25.3	39.0	47	41.9	35.0	41	39.4	43.4	43	41.5	48.5	44	41.6
Flatulence	28.5	89	29.1	36.3	78	26.4	40.7	72	27.0	46.6	73	28.7	43.3	67	29.0
Faecal incontinence	16.9	89	24.2	23.5	78	25.8	30.6	73	27.1	30.1	73	24.9	25.9	67	25.2
Sore skin	34.5	89	31.6	29.5	78	28.9	30.6	72	24.2	28.3	73	27.6	27.4	67	25.9
Stool frequency	12.0	89	17.0	19.4	78	19.3	18.5	73	16.8	18.7	73	16.7	15.4	67	16.0
Embarrassment	7.1	89	17.7	8.2	77	18.9	10.0	73	20.6	16.9	73	27.3	9.0	67	18.9

Supplementary table 2: Mean fatigue score at baseline, and 3 months, 1 year, 3 years and 5 years after chemoradiotherapy.

**Fatigue score**	**Baseline n = 110**		**3 months n =97**		**1 year n= 95**		**3 years n=94**		**5 years n= 82**	
	**Mean**	**SD**	**Mean**	**SD**	**Mean**	**SD**	**Mean**	**SD**	**Mean**	**SD**
Total score	14.6	4.8	17	5	16.3	5.2	16.4	5.5	15	5.8
Physical fatigue	9.9	3.9	11.7	3.8	11.1	3.8	10.9	4.1	9.8	4.1
Mental fatigue	4.8	1.4	5.3	1.8	5.3	2	5.5	2.2	5.2	2.3

Supplementary table 3: Factors associated with total fatigue

A) Univariate

Total fatigue score		Univariate analysis																			
		Baseline				3 months				1 year				3 years				5 years			
		n	Coeff	95% CI	p value	n	Coeff	95% CI	p value	n	Coeff	95% CI	p value	n	Coeff	95% CI	p value	n	Coeff	95% CI	p value
Age	< 70 (ref)	110				97				95				94				82			
	≥ 70		-1.3	-3.6 - 0.96	0.257		-3.0	-5.6 - - 0.5	0.018		-1.9	-4.5 - 0.8	0.159		-2.8	-5.6 - 0.04	0.053		-3.8	-7.3 - - 0.3	0.032
Gender	Female (ref)	110				97				95				94				82			
	Male		-0.7	-2.7 - 1.3	0.470		-1.9	-4.1 - 0.3	0.087		0.4	-1.9 - 2.7	0.724		-1.7	-4.3 - 0.8	0.188		-2.4	-5.3 - 0.5	0.103
Second cancer	No (ref)	110				97				95				94				82			
	Yes		2.4	0.04 - 4.7	0.046		1.2	-1.6 - 3.9	0.402		1.8	-1.0 - 4.6	0.211		0.5	-2.6 - 3.6	0.746		3.4	0.1 - 6.7	0.042
High risk (N+ or T3/T4N0)	No (ref)	110				97				95				94				82			
	Yes		0.99	-0.9 - 2.8	0.290		1.6	-0.4 - 3.7	0.112		0.8	-1.4 - 2.9	0.491		-0.4	-2.8 - 1.9	0.710		1.2	-1.4 - 3.8	0.373
Neuroticism	No (ref)	108				93				89				87				77			
	Yes		3.6	1.5 - 5.6	<0.01		2.9	0.5 - 5.3	0.020		3.0	0.3 - 5.6	0.027		3.7	0.9 - 6.6	0.011		4.4	1.2 - 7.5	0.007

B) Multivariable

		Multivariable analysis														
Total fatigue score																
		Baseline n = 108			3 months n = 93			1 year n = 89			3 years n = 87			5 years n = 77		
		Coeff	95% CI	p value	Coeff	95% CI	p value	Coeff	95% CI	p value	Coeff	95% CI	p value	Coeff	95% CI	p value
Age	< 70 (ref)															
	≥ 70	-1.5	-3.3 - 1.0	0.292	-3.2	-5.7 - -0.7	0.014	-2.1	-4.8 - 0.7	0.136	-3.1	-6 - '1.9	0.037	-4.8	-8.2 - -1.3	0.008
Gender	Female (ref)															
	Male	-1.1	-3.0 - 0.8	0.270	-2.1	-4.2 - 0.1	0.059	0.1	-2.2 - 2.4	0.929	-2.4	-5 - 0.1	0.061	-2.7	-5.4 - 0.1	0.055
Second cancer	No (ref)															
	Yes	1.1	-1.2 - 3.4	0.327	0.4	-2.2 - 3.1	0.745	-0.001	-3.0 - 3.0	1	0.5	2.9 - 3.8	0.783	3.0	-0.5 - 6.5	0.092
High risk (N+ or T3/T4N0)	No (ref)															
	Yes	0.7	-1.1 - 2.5	0.423	1.4	-0.7 - 3.4	0.181	0.8	-1.4 - 3.0	0.468	-0.9	-3.3 - 1.4	0.437	0.976	-1.5 - 3.5	0.435
Neuroticism	No (ref)															
	Yes	3.2	1.1 - 5.3	0.003	2.8	0.4 - 5.2	0.025	2.8	0.1 - 5.6	0.041	3.7	0.8 - 6.6	0.013	3.3	0.2 - 6.4	0.039

Supplementary table 4: Factors associated with chronic fatigue

A) Univariate

Chronic fatigue		Univariate analysis																			
		Baseline				3 months				1 year				3 years				5 years			
		n	OR	95% CI	p value	n	OR	95% CI	p value	n	OR	95% CI	p value	n	OR	95% CI	p value	n	OR	95% CI	p value
Age	< 70 (ref)	68	ref			65	ref			62	ref			60	ref			54	ref		
	≥ 70	18	1.7	0.4 - 6.5	0.460	16	omitted			17	6.5	1.7 - 24.8	0.007	15	2.9	0.8 - 10.3	0.091	7	4.1	0.5 - 36.7	0.204
Gender	Female (ref)	66	ref			59	ref			56	ref			56	ref			49			
	Male	20	1.3	0.4 - 4.1	0.648	22	0.7	0.2 - 2.3	0.553	23	1.1	0.4- 2.9	0.861	19	0.96	0.3 - 2.7	0.943	12	1.3	0.3 - 4.8	0.728
Second cancer	No (ref)	67	ref			67	ref			64	ref			61	ref			49	ref		
	Yes	19	0.6	0.2 - 1.8	0.334	14	0.8	0.2- 3.3	0.758	15	1.1	0.4 - 3.5	0.816	14	1.7	0.5 - 5.8	0.366	12	0.8	0.2 - 2.9	0.752
High risk (N+ or T3/T4N0)	No (ref)	30	ref			33	ref			32	ref			31	ref			22	ref		
	Yes	56	1.3	0.5 - 3.7	0.584	48	0.5	0.1 - 1.6	0.226	47	0.7	0.3 - 1.7	0.411	44	0.9	0.4 - 2.3	0.826	39	1.2	0.4 - 3.6	0.698
Neuroticism	No (ref)	60	ref			57				55	ref			51	ref			41			
	Yes	24	0.6	0.2- 1.8	0.367	20	0.6	0.2- 1.9	0.362	18	0.7	0.2- 2.0	0.458	18	0.8	0.3- 2.4	0.720	15	0.6	0.2 - 2.0	0.394

B) Multivariable

Chronic fatigue		Multivariable analysis																			
		Baseline				3 months				1 year				3 years				5 years			
		n	OR	95% CI	p value	n	OR	95% CI	p value	n	OR	95% CI	p value	n	OR	95% CI	p value	n	OR	95% CI	p value
Age	< 70 (ref)	66	ref			62	ref			58	ref			55	ref			50	ref		
	≥ 70	18	1.7	0.4 - 6.8	0.456	15	omitted			15	9.0	1.8 - 45.3	0.008	14	2.5	0.7 - 9.1	0.158	6	3.9	0.4 - 39.0	0.241
Gender	Female (ref)	60	ref			56	ref			52	ref			51	ref			44	ref		
	Male	24	1.8	0.5 - 6.2	0.366	21	0.6	0.2 - 2.3	0.496	21	1.4	0.5 - 4.1	0.571	18	0.9	0.3 - 2.5	0.769	12	1.1	0.4 - 4.6	0.857
Second cancer	No (ref)	66	ref			63	ref			60	ref			57	ref			46	ref		
	Yes	18	0.7	0.2 - 2.5	0.629	14	0.8	0.2 - 3.8	0.773	13	1.7	0.4 - 6.9	0.455	12	1.3	0.3 - 4.7	0.728	10	0.5	0.1 - 2.5	0.429
High risk (N+ or T3/T4N0)	No (ref)	28	ref			29	ref			28	ref			27	ref			20	ref		
	Yes	56	1.3	0.4 - 4.0	0.601	48	0.4	0.1 - 1.6	0.174	45	0.5	0.2 - 1.5	0.214	42	0.9	0.3 - 2.5	0.834	36	1.2	0.4 - 3.8	0.770
Neuroticism	No (ref)	60	ref			57	ref			55	ref			51	ref			41	ref		
	Yes	24	0.6	0.2 - 1.9	0.394	20	0.5	0.1 - 2.0	0.342	18	0.6	0.2 - 2.1	0.453	18	0.8	0.3 - 2.6	0.761	15	0.7	0.2 - 2.5	0.545

Supplementary table 5: HADS score.

HADS					From baseline to 3 months			From 3 months to 5 years
	Baseline	95% CI	3 months	95% CI	p-value	5 years	95% CI	p-value
**HADS - A**	4.8	3.9 - 5.6	5.0	3.4 - 6.4	0.645	4.2	2.6 - 5.7	0.032
**HADS - D**	3.0	2.4 - 3.7	3.7	2.5 - 5.1	0.023	3.3	2.0 - 4.6	0.154
